# Hematopoietic Stem Cell Transplantation: A Bioethical Lens

**DOI:** 10.1155/2017/1286246

**Published:** 2017-06-27

**Authors:** Arcangelo Liso, Margherita Neri, Francesca Maglietta, Raffaele La Russa, Emanuela Turillazzi

**Affiliations:** ^1^Institute of Hematology, Department of Medical and Surgical Sciences, University of Foggia, Viale Pinto 1, 71122 Foggia, Italy; ^2^Department of Morphology, Experimental Medicine and Surgery, University of Ferrara, Via Fossato di Mortara 70, 44121 Ferrara, Italy; ^3^Institute of Legal Medicine, Department of Clinical and Experimental Medicine, University of Foggia, Ospedale ‘Colonnello D'Avanzo', Viale Degli Aviatori 1, 71122 Foggia, Italy; ^4^Istituto Clinico-Scientifico Malzoni, 83100 Avellino, Italy; ^5^Department of Anatomical, Histological, Forensic and Orthopaedic Sciences, Sapienza University of Rome, Viale Regina Elena 336, 00185 Rome, Italy

## Abstract

Hematopoietic stem cell transplantation (HSCT) is one of a range of therapeutic options available to patients suffering from various diseases. HSCT procedure involves important ethical and legal aspects that can occur at every phase of the procedure: the clinical choice of whether to perform the procedure, pretransplantation preparation regimens, donor selection, stem cell harvest procedure, transplantation phase, and short-term and long-term follow-up care. In this discussion paper, we outline the ethical issue-facing physicians involved in HSCT. Currently, HSCT is a widely accepted treatment for many life-threatening diseases. It thus represents a real therapeutic hope for many patients. It does, however, carry a burden of possible morbidity and mortality. Consequently, there are substantial information and communication issues involved in the consent process for HSCT. In the final decision, the judgements of different parties, such as patients, family members, and healthcare professionals, intersect and overlap and this is particularly true when the patient is a minor. Finally, HSCT is a very expensive procedure. The social and economic concerns of HSCT are discussed within the actual contextual framework of the dramatic increase in healthcare costs and inequalities in healthcare in relation to socioeconomic status, educational status, and ethnicity.

## 1. Introduction

Hematopoietic stem cell transplantation (HSCT), either autologous or allogeneic, is one of a range of therapeutic options available to patients who suffer from refractory or relapsing neoplastic disease and nonneoplastic genetic disorders, as well as from chronic bone marrow failure. In fact, over the last few decades, considerable progress has been made with regard to HSCT because of major discoveries in basic science, improved diagnostic procedures, and newer therapies. Many patients have already benefitted from this form of treatment, and the encouraging results obtained are well known [1, 2].

HSCT is a very complex medical procedure which has led to the development of groups of specialized healthcare professionals with similar scientific interests and common professional standards. In turn, this has also led to the production of specific guidelines (e.g., on fertility preservation and donor algorithm selection) [3–6].

Guidelines are important not only for the management of medical issues but also for the HSCT procedure because it involves important ethical and legal aspects [7, 8]. Ethical problems concerning transplantation can occur at every phase of the procedure: the clinical choice of whether to perform the procedure, pretransplantation preparation regimens, donor selection, stem cell harvest procedure, the transplantation phase (including hospitalization during marrow aplasia), and short-term and long-term follow-up care.

Moreover, since HSCT is also clearly associated with a substantial risk of life-threatening acute complications as well as significant organ toxicity, the quality of life during and after HSCT, compared to that associated with alternative treatments, is a very important issue which should be carefully discussed and evaluated with the patients.

In this discussion paper, we will outline bioethical issue-facing physicians involved in HSCT and the wider scientific community, with a particular focus on the difficulties in defining an adequate consent process.

## 2. A European Overview

The number of HSCT-treated patients has significantly risen over the last two decades, with estimates of 45,000–50,000 procedures performed each year worldwide [9]. In a global perspective, more than 14 million typed volunteer donors or cord blood units from the many registries worldwide provide stem cells for patients without family donors [10, 11].

A recent retrospective study [12] on behalf of the Worldwide Network for Blood and Marrow Transplantation (WBMT) showed that 953,651 HSCTs were registered from 1516 transplant centres in 75 countries. No transplants were performed in countries with fewer than 300,000 inhabitants, a surface area of less than 700km^2^, or a gross national income per person of US$1260 or lower. As expected, transplant rates were higher in countries with more resources, more transplant teams, and unrelated donor infrastructures [12]. Countries with major socioeconomic concerns have the lowest HSCT performances: Latin America numbers are lower than those in North America and in European regions but higher than those in the Eastern Mediterranean and Asia Pacific regions [13, 14].

In Europe, an increase in the annual absolute HSCT numbers and transplant rates has been reported [15]. However, also in Europe, economic factors, for example, gross national product per capita, healthcare expenditure per capita, type of healthcare system, and income in general, strongly condition the transplant rates for each country [16, 17]. Passweg et al. [15] report that in some evolving European countries (i.e., Eastern Europe), the increase in HSCT is particularly noticeable.

The field of tissue and cell donation and banking is now highly regulated in many countries. According to the World Health Organization (WHO) Aide-Mémoire on the donation and transplantation of tissues and cells, national health authorities are responsible for ensuring that the donation, banking, and human application of tissues and cells are promoted, regulated, and monitored appropriately in the interests of patient safety and public transparency. More specifically, they are responsible for ensuring that (a) an appropriate legislative/regulatory framework is in place; (b) national/international practice standards have been defined; (c) there is inspection/authorisation of screening, testing, procurement, processing, storage and distribution, imports, and exports; (d) there are programmes for vigilance and surveillance of adverse outcomes; and (e) there is monitoring and reporting of donation, processing, storage, distribution, and import and export activities.

The need for the international standardization of cell and tissue banking practices has been felt in Europe since 1978. In that year, the Council of Europe adopted Resolution (78) that assessed the harmonization of legislation relating to the removal, grafting, and transplantation of human substances. Over the following years, several European Union (EU) Directives have stated the requirements which have been (or are in the process of being) transposed into the national legislation of the EU member states. Issues related to the safety and quality of HSCs are regulated by European Directives 2004/23/EC [18], 2006/17/EC [19], and 2006/86/EC [20].

These directives are legally binding for member states. In addition, the guide to the quality and safety of tissues and cells for human application was issued by the European Committee (Partial Agreement) on Organ Transplantation (CD-P-TO) in 2013 and is in its second edition. This guide contains the instructions considered to be the “minimum standards” that align with relevant EU Directives in the field and provides assistance for those states outside the EU that are considering adopting the EU requirements in their legislation.

Furthermore, international standards for HSCT have been developed in Europe by the Joint Accreditation Committee of the International Society for Cellular Therapy (ISCT) and EBMT (JACIE), the European equivalent of the US Foundation for the Accreditation of Cellular Therapy (FACT) [21].

The report on the first ten years showed that 200 European programmes have applied for JACIE accreditation, which has been granted in more than 100 cases. The report identified the status of the transplant programme regarding JACIE accreditation as the major factor affecting the overall survival of recipients of allogenic transplantation [22, 23]. Although over the years almost all of the inspected European centres were found to be functioning at a high level of excellence [24, 25], a challenging step forward relates to collaborative projects to implement the adoption of JACIE standards as reference for the regulatory accreditation process, as it already occurs in some European countries [26].

## 3. Medical and Economic Issues

Recent progress in several areas of cancer therapy (e.g., the successful treatment of some hematopoietic malignancies) has been quite remarkable, with cure rates of up to 80–90% for certain diseases and in selected patient groups [27]. However, recently, there has also been a dramatic increase in healthcare costs, and both insurance programmes and national health systems are facing growing demands on resources [28]. Reasons for this phenomenon include the following:

- The costs related to prolonged and recurrent hospitalization
- The availability of multiple treatment options (including HSCT) which are not mutually exclusive and therefore can be used sequentially
- The price of new medications (e.g., specific inhibitors, monoclonal antibodies, and recombinant molecules)
- The aging of the population in Western countries.

In fact, the total number of cancer diagnoses and cancer survivors is increasing [29] and growing numbers of older individuals are receiving anticancer therapy which is often intensive. Only intensive chemotherapy followed by hematopoietic cell transplantation is generally considered potentially curative in neoplastic diseases.

HSCT is a very expensive procedure; however, it has the clear advantage of potentially eradicating the neoplastic clones. Indeed, the field of HSCT has made major progress in the treatment of many conditions and has also proved that stem cell therapy and immunotherapy are effective against malignancies. The success of HSCT is derived both from the ability to treat patients with intensive chemoradiotherapy and, in some diseases like leukaemia, from potent graft-versus-leukaemia (GVL) effects mediated by donor immune cells. Importantly, HSCT has been a curative therapy also for several nonmalignant hematologic disorders.

Notably, progress over the years has been remarkable in understanding histocompatibility, graft-versus-host disease (GVHD), GVL effect, and immune reconstitution after transplant. The development of unrelated donor registries and increased utilization of cord blood and partially matched related donor transplants have all ensured that there is a donor for nearly everyone who needs a transplant. Moreover, improved donor selection, patient-tailored conditioning regimens, and better supportive care measures have helped reduce morbidity and mortality.

On the other hand, significant obstacles are disease relapse, infectious complications, and regimen-related toxicities. It is important to note that comorbid conditions also interfere with effective therapy. Furthermore, intensive chemotherapy-based conditioning regimens in older individuals may be associated with considerable morbidity and the need for prolonged hospitalization and rehabilitation, thus stressing the system and draining family resources. Although transplantation following low-intensity conditioning is being carried out in patients even in their 70s, these are mostly highly selected patients, and the data generated in clinical trials are difficult to extrapolate to the population at large [30].

To make things even more complicated, in some patients, HSCT-related complications are superimposed onto preexisting chronic organ insufficiency, such as renal insufficiency related to previous nephrotoxic treatment. Notably, almost all patients entering the HSCT procedure show significant immunosuppression, either because of cytostatic or immunosuppressive pretreatment or as a result of their underlying disease.

As the focus of many older individuals is on the quality of life, it is important to emphasize that, for various advanced malignancies, emerging data indicate that the quality of life may be better and survival may be longer with nontransplant procedures and/or with palliative care.

As an example, the management of chronic myeloid leukaemia (CML) has dramatically changed over the past few years [31]. HSCT was the standard of care for eligible patients until the 1990s. Despite the fact that considerable results were obtained in those patients, better knowledge of the molecular basis of CML led, in the late 1990s, to the development of tyrosine kinase inhibitors (TKIs) which revolutionized the management of the disease. TKIs are a tool for achieving long-term disease control with simple oral therapy and are now the first-line treatment. The majority of patients treated with TKIs achieve excellent responses with sustained treatment, and some even continue to have exceptionally low-level disease after TKI withdrawal. Therefore, only to those with an inadequate response to any of the currently available TKIs, should HSCT be offered as the best option for achieving long-term survival.

Finally, a reassessment of treatment decisions in some patients is probably needed. Medical doctors involved in HSCT teams probably need not “oversell,” and particularly older patients may not have a full understanding of the impact of the proposed therapy on their lives. Our conversations with these patients must include a discussion of alternatives and supportive care and, even more importantly, must address end-of-life issues sand expectations.

## 4. Social and Cultural Issues

There is some evidence of inequalities in healthcare in relation to socioeconomic status, educational status, and ethnicity [32–34]. Moreover, migrants are often more disadvantaged than the indigenous population because they have moved from a poor environment, have a lower educational status, and may have little understanding of the language. As most migrants come from places with poor healthcare facilities, they may have underlying health problems that have been inadequately addressed over the years [35]. Furthermore, since migrants have attitudes to healthcare associated with their own culture, they may respond less well to preventive care opportunities such as vaccination. In fact, many populations have a distinct perception of acute illness but have no understanding of subclinical/asymptomatic chronic diseases. Without an understanding of how to manage chronic problems, patients may think that medications should be discontinued as soon as symptoms are no longer present, and this can be detrimental when long-term care is needed (e.g., preventing GVHD). In addition, each exacerbation of symptoms may be seen as a separate illness rather than, for example, the long-term side effects of HSCT, especially if there are several symptoms. It is also likely that remission will be understood as a definitive cure, and this is particularly important to avoid following HSCT, when the diagnosis of early relapse can be of vital importance, as in acute leukaemia cases.

Finally, migrants are occasionally unfamiliar with healthcare practices in their host country (such as appointments and registration procedures) that are important for successful clinical management in the long-term follow-up after HSCT. Overall, racial and ethnic minorities are less likely to receive even routine medical procedures and more complex transplantation procedures. The relationship of immigrants with their physicians is as important as for other patients. Communication is an important part of healthcare, and immigrants' difficulties with the language of the host country may cause difficulties in medical investigation, lack of recognition of mental illness, and poor compliance with therapeutic recommendations [36].

Immigrants' individual beliefs about health and illness are culturally determined and may affect health, self-care practices, the type of healthcare sought, and the degree of compliance. Beliefs are essential for self-care practice and care-seeking behaviour and must be considered when planning clinical care and when offering HSCT.

Modern approaches to healthcare give the patient an active role and characterize the relationship between the physician and the patient as a partnership. Immigrants, on the other hand, are mostly inclined to accept illness as part of their fate (due to superstition), and they have difficulty discussing their problems with a medical advisor in a manner that would give them an active role in their health management. More common in their culture, perhaps, is having a doctor tell them what to do. Their fear of an inability to work and its inevitable economic consequences for the welfare of their families dominate their behaviour during consultation and when advised to undergo a complex procedure like HSCT which implies long periods of hospitalization.

Finally, the vast majority of doctors have recently been facing the problem of patients taking complementary and alternative medicines, which clearly play no role in HSCT. This can hamper communication, especially in cross-cultural encounters. Recent immigrants and refugees who are unfamiliar with Western healthcare are far less likely to share such information. They may feel Western doctors will not understand or approve of their culturally based remedies, and they fear being judged for their “foreign” beliefs. They may not be aware that preventing adverse drug interactions depends on discussing all the medicines they take with their doctor.

## 5. Bioethical Issues

Organ donation and transplantation has become gradually more and more critical in the field of medicine, and it is therefore of vital interest to society and law. As medical knowledge advances, with scientists and investigators discovering numerous therapeutic measures, organ donation and transplantation has become indispensable, since the progress of modern medicine enables a growing variety of possible transplantations.

However, in organ donation and transplantation, donors' and recipients' priorities may conflict and create ethical problems. Primary emphasis has thus to be placed on the balance between potential risks and benefits for both the donor and the recipient and the maintenance of a therapeutic dimension of the entire donation/transplantation process. When we shift to a broader vision that also invests the social dimension of the phenomenon, the gap between availability and need becomes a central knot [37]. As with organs, the demand for some transplantable tissues and cells far outweighs the available supply. Individual motivation and choice is only one part of the donation picture; the central role of organizations, organizational procedures, and professionals in facilitating donation should be highlighted.

In general terms, difficulties in donating organs may include lack of knowledge and misperceptions about organ donation, difficulty obtaining familial consent in organ donation from the deceased, and insufficient infrastructure. The personal ethical values, beliefs, and religiosity of the potential donors may intersect with structural and organizational barriers to explain the lack of organ availability.

In this scenario, HSC donation presents concerns that are common to other organ and tissue donations: cultural and personal values potentially affecting the decision to become an HSC's donor [38–41] and mistrust either of the medical system at large or in the equitable allocation of HSC donation [38, 41–45]. Satisfaction with the decision to donate or, in contrast, fear of donation and of potential health consequences may be other conditioning factors in all living organ and tissue donations [46–48].

HSC donation presents particularities in comparison with other types of tissue or organ donation that can affect the donation rate [49]. It can be said that HSCT can be situated on the middle ground between blood donation and living organ donation [49]. However, HSC donation carries many more risks and inconveniences compared to blood donation. It may require the use of drugs such as the granulocyte colony-stimulating factor (G­CSF) with the aim of harvesting enough stem cells from peripheral blood or minor surgery and anaesthesia in bone marrow donation. On the other hand, undoubtedly, HSC donation is less burdensome, both physically and psychologically, compared to the donation of organs (e.g., kidney and liver) among living subjects.

A peculiarity of HSCT, perhaps even more than for other types of transplant, is that human leukocyte antigen (HLA) matching is of paramount importance, and it is the major donor-related factor in the success of HSCT [50–52]. Furthermore, in HSC transplantation, repeated donations may be necessary [53]. Consequently, there may be quite a long time interval between the moment the potential donor decides to donate and enrols in a register or centre and the donation actually taking place [49, 54]. It has been reported that the time between enrolment and actual donation is, on average, eight years [49]. Thus, a problem peculiar to unrelated HSC donation is the long gap between the first motivation to join a donor registry and the final act of donation, usually several years later.

Furthermore, younger age groups seem to correlate positively with the intention of donating HSCs [55]. These elements should be taken into account, since potential donors may change their mind over the years and may refuse donation when the time comes. In the same way, repeated donation may be refused.

Finally, HSCT is a complex, high-cost procedure and depends on a well-established institutional infrastructure network [56].

In conclusion, similarities between HSC donation and other types of living tissue and organ donation exist; however, given the particularities of HSC donation, specific strategies to increase donation can be undertaken.

The creation and implementation of stem cell banks and registries is an important step towards increasing HSC donation since these organisms may play a pivotal role in recruiting, registering, and coordinating more potential donors. HSC registries may serve as hubs, linking donors, recipients, clinics, biobanks, and regulatory agencies [57–60] and are thus a useful tool also in promoting a donation culture. Providing accurate information and knowledge about stem cell donation remains a priority in increasing the number of donations. Recent studies have demonstrated that also educational and communicative efforts towards the medical community may be beneficial in the recruitment and retention of donor populations [61, 62]. The creation of a social and cultural atmosphere favouring the diffusion of correct and proper information regarding HSC donation is central in encouraging people to make a donation. Furthermore, the motivation enhancement strategies employed by major societies and agencies are critical in increasing HSC donation. With particular reference to HSC donation and due to the long time lapse between the decision and actual donation, the standards of the World Marrow Donor Association (WMDA) assess the donor's right to withdraw even if this might entail serious and even fatal consequences for the recipient. At the same time, they reiterate the importance of the potential donor's full understanding of the serious and potentially life-threatening consequences to the recipient if the donor chooses to withdraw at any time, but particularly once the recipient's pretransplant conditioning has begun [63, 64]. Finally, a more effective promotion of HSC donation, which has traditionally relied on interpersonal communication and the mass media, is of paramount importance in ensuring the sustainability and continuity of donation activities. The debate surrounding advertising and solicitation is still unresolved [65–67]. As for the living donation of other organs and tissues, the use of media and other forms of communication to advertise the need for organs or tissue for oneself or for a related person can be considered a universal right. Undoubtedly, patients and their families have the right to tell their own stories publicly through any information channel, and personal stories by the family members and friends of patients often result in increased media interest and can be used to communicate the importance of registration and donation to any patient that could need it.

On the other hand, the increase in highly publicized appeals for organ and tissue donation for individual patients may give rise to concerns. First of all, it may be counterproductive to the needs of many others requiring organ transplantation, having a potential direct or indirect impact on less-fortunate or less-connected families. Furthermore, both the correctness of information and the emotional pressure on potential donors have to be carefully taken into account.

In conclusion, given that there is a stringent need to improve HSC donation, several strategies may be undertaken to encourage people to donate. The creation and implementation of banks and registries and the diffusion of a social and cultural atmosphere of donation through information campaigns are pivotal to motivate people to donate. Other strategies, such as advertising and solicitation, are still debated and may be ethically acceptable as long as certain conditions, such as correct information to potential donors, are maintained [49].

When dealing with the bioethical issues related to HSCT, the issue of informed consent is a very central one. Adults with decision-making capacity have a long-recognized and legally protected right to make decisions about their bodies and health. Consent is a basic ethical consideration, implying that it is informed and that patients must be given adequate and accurate information. HSCT is a “high-stakes” medical treatment [68]; people who are undergoing HSCT should thus be prepared to participate in decisions that involve weighing benefits, harm, long-term risks, and uncertainty linked to the treatment itself [69].

From a general point of view, patients are increasingly involved in decision making related to the nature of medications that reflect deliberative, personal choices. The literature is full of contributions on the subtleties of informed consent, thus finally reinforcing the ethical, legal, and deontological requirements of the patient's informed consent before undertaking any medical procedure [70, 71].

Informed consent gives the patient the power and the right to choose to opt out of/in the treatment. However, a number of troubling issues cluster around the patient's decision on HSCT [8] since, in the final decision, judgements made by different parties, such as patients, family members, and healthcare professionals, intersect and overlap.

The first step is to assess the medical indications for and the clinical effectiveness of HSCT. Is it reasonably expected to benefit the patient? Are there predictable side effects? Are these tolerable in the light of the expected benefits? As HSCT is a high-risk procedure [72], these points are critical in forming the judgements of both the physician and the patient. However, the perception of risks relating to a medical procedure is complex even for physicians [73] and the magnitude of risks is only one of the several factors that may influence it. Other factors that may contribute to risk perception by a patient include perceived fear, the potential severity of the risk, and how familiar or unfamiliar the risk is. Some risks, therefore, may be more unacceptable than others that are more familiar and perceived to be more controllable for a single patient [74]. For example, some patients may place greater emphasis on the potential for cure of their disease and less importance on possible treatment-related side effects that may develop, while other patients may have opinions which are exactly opposite [75]. It has been reported that overestimating the benefit of transplant is evidenced by the fact that patients rate the perceived success of a cure after HSCT as being 78% higher than their physicians do [76].

Furthermore, once the medical indication for HSCT is established on the basis of sound science, patient preferences remain essential in the light of the recommended medical treatment, since HSCT involves several different aspects which include domains related to emotional, physical, mental, social functioning, and quality of life, potentially impacting compliance to HSCT treatment. Patients' perceptions and expectations are substantial in the decision-making process, since it has been shown that the experience of HSCT may have a wide, long-lasting, profound impact on recipients [77, 78]. Lee et al. explored the discrepancies between the patients and their physicians regarding HSCT and found that, in this clinical setting, patients and their physicians had the most concordant expectations when the outcome of HSCT was likely to be good, while patients with more severe disease failed to recognize the higher risks associated with their clinical condition [74]. For example, a clear association between actual survival and the physician's estimates, but not the patient's estimates, was observed in a total of 123 patients and their attending physicians by Grulke et al. [77]. Such a situation reveals the complexity of the decision in the specific setting of HSCT as a dispute may occur when the patient's preferences are contrary to the recommendations of the physician. The physician, wishing to provide a benefit to the patient, offers HSCT. When the patient refuses the medical recommendations even if the proposed treatment may be lifesaving, the situation demands a positive response following the bioethical principle of autonomy [79]. However, a full rendering of the meaning of autonomy implies that the patient's capacity for such a decision is not compromised: the physician has provided all the facts about the patient's disease, prognosis, and therapeutic alternatives, and the patient has assessed each alternative treatment in terms of his/her own value. Patients with hematologic malignancies, for whom HSCT is recommended, have to deal with the diagnosis of a threatening disease that brings feelings of sadness and anger as well as uncertainty about the success of the treatment. Sometimes, the problem may centre on different ideas about an acceptable quality of life that often come into play in the decision-making process. The physical and psychological burdens experienced by patients undergoing HSCT are well known [80] and may have short- and long-term quality of life (QOL) consequences, further exacerbating the morbidity of HSCT [81–83]. HSCT has a profound and pervasive impact on the lives of survivors [84]; in a cohort of five hundred and ninety patients from six transplant centres, Valkova et al. recently demonstrated that patients with a GVHD had an inferior QOL score and that QOL decreased with increasing age and increased with time elapsed since HSCT. Despite the fact that QOL increases steadily as more time elapses since HSCT, sometimes, the impact of the consequences of HSCT may persist permanently, thus being a substantial factor in the decision-making process by patients [83].

In the final analysis, consent to HSCT is a complex situation-specific, value-laden, and goal-dependent process which may best be made only after complete information has been processed by the patient to allow a fully informed decision. It is affected by many different factors such as the value systems of both physician and patient, medical goals, clinical effectiveness, sociocultural and religious context, and the individual's emotions and personal characteristics.

All these ethical and legal challenges are even greater when HSCT involves the paediatric population.

The application of HSCT in the case of children and adolescents is vast and growing and includes several malignant and benign diseases which are incurable by any other therapies [84, 85]. The successful treatment of many paediatric diseases with HSCT has resulted in an increased number of long-term survivors, thus increasing concerns about long-term adverse effects (e.g., GVHD, opportunistic infections, future infertility, developmental delay, and secondary malignancies) [86, 87].

Paediatric patients who are also HSCT recipients generate unique concerns which are both clinical and ethical. The basic assumption is that, in the paediatric population, there is a tripartite relationship between the child, parents or surrogates, and clinicians [88]. Furthermore, the paediatric population includes subjects of different ages, ranging from neonates who are completely excluded from the decision-making process to late adolescents who are partially able to make a decision [88]; thus, the value of involving children and adolescents in their own medical decision making is increasingly recognized [89].

Open communication with parents and families should be the gold standard of the physician-parents-patient relationship. The physician and the parents each have their own responsibilities and concerns: those of the physician are technical and scientific while those of the parents relate to pain, perception, and the quality of life. Yet all cooperate in resolving the health problems of the sick child. Parents are active participants in a dialogue that is important in the shared decision-making process; the physician has the burden of ensuring that comprehension is achieved and that the parents themselves are acting in the best interests of the child. Parents, physicians, and, when possible, the paediatric patient must consider and balance the risks of graft rejection, infection during transplantation, immunosuppression, GVHD, and death against the potential benefits of HSCT when deciding about HSCT. In their studies on this issue, authors have shown that a substantial proportion of adult patients and parents of children with sickle cell anaemia requiring HSCT is willing to accept a certain amount of risk in the hopes of curing their disease and living a normal life [90–92] with the highest levels of acceptance related to the increased severity of the disease. However, there continue to be significant numbers of parents and adolescents who are unwilling to accept any risk of HSCT-associated mortality or GVHD [93].

The issue is decidedly more problematic when a mature minor and his/her parents find themselves in disagreement about whether or not to undergo HSCT. Mature minors, that is, those able to understand the nature and the eventual burdens of a medical treatment, may express strong desires to know about their clinical condition. These can be extremely difficult concerns for the adolescent to consider and balance, because their decision includes the need to consider both acute and long-term risks and benefits. Refusal of life-sustaining medical treatment such as HSCT may result. Whether the right to refuse life-saving therapies applies to minors (typically defined as younger than 18, though the definition varies according to the country) is very complex. Generally, the legal norm for minors is that parents provide consent on behalf of the child and the child provides “assent” to the extent that he or she is developmentally able to do so. The issue of the refusal of life-sustaining treatment by mature minors is still strongly debated [94–98], and the ability of children to refuse medical treatment is far from certain [99].

Even if the right to refuse medical treatment is likely to be restricted to those who are competent, physicians cannot just shrug it off. Refusal of life-sustaining therapy such as HSCT by a mature minor should be given careful consideration by physicians and parents [88]. There are some difficult problems here that should be addressed. Physicians have the duty to assist all the family members, including the adolescent patient, involving other members of the multidisciplinary team, in order to reach a shared decision consistent with their beliefs and values. Consideration must be given to the interests of all parties. However, respect for autonomy, self-determination, and the best interests of the minor must always be at the forefront. Legal advice may be helpful in deciding whether application should be made to the court to resolve disputes about best interests that cannot be resolved informally. A statement from the Confederation of European Specialists in Paediatrics clearly states that paediatric patients may not refuse life-saving treatment.

Conclusively, the concerns surrounding informed consent, which is one of the most challenging in HSCT, become amplified when the subjects involved are children. The practical understanding of children and parents of goals and procedures, risks, future implications, and, finally, alternatives to HSCT is the critical key point of the informed consent process. A balance between parental permission and child involvement in the decision-making process strongly depends on the age or maturity of the child and on the degree of communication between parents and children. Broadly, the scenario of consent to HSCT may vary from a choice mostly based on the parent's decision in the case of very young children, to a joint decision as children mature, and, finally, to a largely independent decision made by an older adolescent with parental affirmation.

## 6. Conflicts of Interest

Conflict of interest has emerged as one of the most serious ethical problems the international community faces [100]. In fact, this is an issue that affects the very dignity and prestige of medical science as a whole but, obviously, also the wellbeing of patients. Today, this conflict can manifest itself in a number of specific ways [101]. Firstly, it can arise between economic interests on the one hand and medicine and healthcare on the other. Most biomedical research has been predominantly carried out in and for developed countries. This is reflected in the fact that the WHO has estimated that the vast majority of the resources devoted to research and development on medical problems are applied to diseases that affect a minority of the world population. Secondly, it can be seen in the selection of research programmes which are likely to give quick profit, whereas research that involves higher costs and greater investment of time (i.e., HSCT) can be excluded [102].

Another example of conflict of interest in HSCT is the permission to publish research data from the sponsoring groups which can therefore selectively choose what to publish. Finally, the need for recruiting large numbers of patients in transplant research centres may induce physicians to propose the HSCT procedure in a convincing way to prospective patients, rather than possible therapeutic alternatives. For example, an important ethical issue has been the implementation of autologous HSCT in clinical practice on the basis of phase-II trials in the 1990s and the publication of falsified data in the first randomized controlled trials [103].

Public authorities play an active role in ensuring that research is directed towards increasing the standards of healthcare in the interests of people and of society and in tempering and reconciling the pressures of different interests. Ethical and scientific standards for carrying out biomedical research on human subjects have been developed and established in international guidelines [7] including the Declaration of Helsinki, the CIOMS (Council for International Organizations of Medical Sciences) International Ethical Guidelines for Biomedical Research Involving Human Subjects, and the World Health Organization and ICH (International Conference on Harmonization) Guidelines for Good Clinical Practice. Compliance with guidelines helps to ensure that the rights, safety, and dignity of research participants and that the results of the investigations are trustworthy. All international guidelines require ethical and scientific review along with informed consent (and the appropriate protection of those unable to consent) as essential measures to protect persons who participate in research.

While the scientific review is handled by scholars, the very purpose of an ethical committee (EC) in reviewing biomedical research is to contribute to safeguarding the rights, safety, and health of all actual or potential participants in the research programme. Therefore, the people involved in the ECs come from diverse backgrounds and are typically lawyers, patient advocacy groups, public health experts, professors of bioethics, an so on.

ECs should also take the principle of justice into consideration so that the benefits and risks associated with a new research programme can be fairly distributed among all groups in society, irrespective of gender (when appropriate), economic status, and ethnicity.

Finally, ECs should provide an independent and competent review of the ethics of proposed studies. Both in the procedures and in decision-making, ECs need to be independent of political and market influences.

ECs are responsible for carrying out the review of the proposed research before it is begun, so timely evaluation is of crucial importance. They also need to ensure that there is a regular evaluation of the ethics of ongoing studies that received a positive decision, as interim results can be illuminating on the efficacy and side effects of new protocol.

In summary, ECs are responsible for acting in the interests of research participants, also taking into account the interests of the researchers, and the requirements of relevant regulatory agencies and all applicable laws.

## 7. Conclusions

When discussing ethical and legal issues in HSCT, there are many controversial areas. This paper offers a brief description of some questions, but it is neither complete nor comprehensive. Hopefully, it may serve to stimulate the discussion of such themes within the medical community, involving also the society at large.

As HSCT practices increase, the challenges are great. Some of the ethical issues will be worked out in the privacy of the physician-patient relationship. Medical indications for HSCT, the likely success of HSCT, the patient's preferences and expectations, his/her quality of life, and the patient's contextual features are the core of the decision-making process, representing the real relevant facts pertaining to the situation into account. All these issues are decidedly more problematic when they concern a patient who is a minor. At the same time, however, the concern of physicians should be for the good of society and the financial sustainability of the healthcare system, thus extending benefits as equitably as possible.
